# Examining the antecedents and consequences of mobile travel app engagement

**DOI:** 10.1371/journal.pone.0248460

**Published:** 2021-03-12

**Authors:** Zemin Tian, Zhangyu Shi, Qian Cheng

**Affiliations:** 1 School of Tourism and Urban-rural Planning, Zhejiang Gongshang University, Hangzhou, Zhejiang, China; 2 Hangzhou College of Commerce, Hangzhou, Zhejiang, China; Aalborg University, DENMARK

## Abstract

How and why customers engage with mobile travel apps is vital to mobile marketing of travel-related companies. This paper discusses the antecedents and consequences of mobile travel app engagement. Specifically, this study aims to understand how travel app attributes stimulate mobile travel app engagement and lead to purchase intention. A research model is established based on the Stimulus-Organism-Response (S-O-R) model and the model is tested by Partial Least Squares Path Modeling (PLS-PM). The results show that ease of use, compatibility, and UI attractiveness positively influence mobile travel app engagement, and in turn, affect purchase intention. Furthermore, a multi-group analysis shows that the attributes affecting mobile travel app engagement differ across different customer groups. This paper discusses some theoretical and practical implications.

## 1 Introduction

“Mobile apps” are software applications installed on devices such as smartphones to allow users to perform specific tasks or execute certain functions [1]. The mobile app market has experienced tremendous growth alongside advancements in mobile phones, tablets, and other smart devices. Today, mobile apps are an indispensable part of many people’s lives [2]. Mobile apps are utilized for communication, shopping, news, education, travel, and a wide array of other purposes.

The mobile Internet is not limited by time or space in same manner as home Internet resources, which makes it a natural fit for the tourism industry. Mobile travel apps are “specifically targeted at travelers” [3] to allow them to search for and book accommodations, tour activities, and flights on-demand [4]. These advantages make mobile travel apps very popular among tourists. According to a report released by the China Internet Network Information Center, by December of 2018, the number of users who book travel products through mobile devices had reached 392 million accounting for a total of 49% of travelers [5].

Many scholars have studied the factors that influence consumer adoption of mobile travel apps [6–8]. Previous researchers have found that consumers are willing to download mobile apps, but go on to delete about 50% of them from their devices [9]. So, how do travel-related companies increase mobile app retention and improve these online conversion rates?

“Customer engagement” refers to customers’ motivational experiences when connecting with media channels or brands [2]. To better study mobile travel app engagement, it is necessary to characterize the differences between adoption, continuous usage, and app engagement. App adoption behavior and app engagement are conceptually different; app adoption is the starting point of app engagement [2] while app engagement is a continuous process of interacting with app post-adoption. In addition, although both continued usage behavior and app engagement reflect users’ post adoption behavior, they have disparate respective definitions.

Continued usage behavior is often regarded as a process where by individual consumers utilize a product continuously and long-term after adoption [10]. App engagement refers to users’ continued interaction with a product [11]. Customer engagement is a multi-dimensional concept which generally consists of cognitive, affective, and behavioral elements of individual experiences [12–15]. Thus, continuous usage is a subset of behavioral app engagement. Little is known about antecedents and consequences of mobile travel app engagement, specifically. Considering that mobile travel app engagement is essential in terms of travel-related companies’ relationship marketing effectiveness, information regarding customer engagement with mobile travel apps has important implications for strategic management.

The main purpose of the current study is to propose a model to explore the effects of travel app attributes on mobile travel app engagement, which subsequently affects purchase intention. The secondary purpose is to assess whether certain attributes of different customer groups affect mobile travel app engagement and purchase intention differently. The findings of this study may help travel-related companies guide their relationship marketing strategies to foster customer engagement and promote customer retention.

## 2 Theoretical model and hypotheses

Mehrabian and Russell [16] first proposed the Stimulus-Organism-Response (S-O-R) theory, which serves to reveal the manner in which people respond to external environmental stimuli. S-O-R theory suggests that external stimuli (S) trigger internal reactions (O) that lead to certain responses (R). The S-O-R theory has been applied to information systems field [17,18]. In this study, the S-O-R model allowed us to explore the relationship between travel app attributes, mobile travel app engagement, and purchase intention.

**Fig 1:**
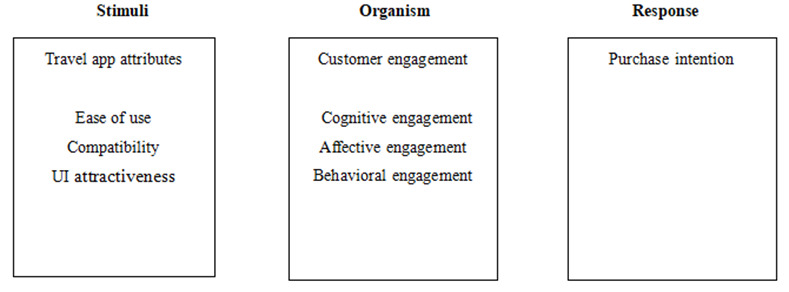
S-O-R model.

Drawing on the S-O-R model, we propose that attributes of a travel app (stimuli) may influence customer engagement with mobile travel apps (organism) in turn affecting purchase intention (response). The proposed S-O-R model is shown in Fig 1.

### 2.1 Customer engagement

At present, there is no consensus regarding the customer engagement concept; different scholars have defined it from various perspectives. The opinions of scholars can be roughly divided into three categories. The first category contains opinions from the perspective of motivation. Algesheimere et al. [19] defined customer engagement as the intrinsic motivation for customers to interact and cooperate with community members. From a behavioral perspective, Van Doorn et al. [20] defined customer engagement as “a customer’s behavioral manifestations that focus on a firm or brand, beyond purchase, resulting from motivational drivers”. However, consumer engagement can also be characterized as a psychological process that evolves from an ongoing experience with a focal agent or object [12].

Customer engagement is considered a multi-dimensional concept, generally including cognitive, affective and behavioral elements of the individuals’experiences [12–15]. Many variables influence consumer brand engagement, particularly within online brand communities [13–15,21]. Engagement is interactive and context-dependent [12,22], so the variables influencing consumer engagement may differ across different contexts. It is necessary to fully understand the factors affecting customer engagement in the unique context of the mobile app.

### 2.2 Travel app attributes

Previous studies have shown that product attributes are critical external stimuli influencing individuals’ internal cognition and emotions [23]. Fang et al. [24] assert that the performance attributes and design attributes of an app are the main factors that influence mobile app engagement. Software quality, in general, is comprised of functional quality and structural quality indicators [24]. App performance attributes reflect the extent to which an app fulfills its functions as expected. App design attributes reflect the structural quality of the software itself. Ease of use and compatibility are the main attributes of app performance and UI attractiveness is one of the key attributes of app design [24]. In this study, we mainly regard ease of use, compatibility, and UI attractiveness as the framework within which to explore the influencing factors of customer engagement with a mobile travel app.

#### 2.2.1 Ease of use

The ease of use of a technological system plays a critical role in the adoption and usage of that system [25,26]. The Technology Acceptance Model (TAM) can explain why individuals adopt particular technologies [27]. Davis [28] suggests that “ease of use” is the extent to which an individual perceives that a technological system can be utilized without effort. In this study, we define ease of use as the extent to which an individual perceives the mobile travel app to require little or no effort.

Product functions that are excessively complex are difficult for customers to navigate, which can lead to a poor experience with the product and reduce the user’s overall satisfaction [29]. A high level of customer satisfaction towards a travel app increases the customer’s psychological engagement with the app [30]. When customers can easily use a travel app, they perceive greater control over the experience which improves their satisfaction and further promotes psychological engagement [31]. Previous research indicated that ease of use affects customer engagement with mobile apps [27]. We propose the following hypotheses accordingly.

H1a. Ease of use is positively related to cognitive engagement with a mobile travel app.
H1b. Ease of use is positively related to affective engagement with a mobile travel app.
H1c. Ease of use is positively related to behavioral engagement with a mobile travel app.

#### 2.2.2 Compatibility

Compatibility is defined as the extent to which innovation is consistent with the consumer’s values, past experience, and current needs [32]. A mobile travel app provides many functions to meet its users’ needs. The app may use, for example, location-based information in order to provide tourists with tailored recommendations for attractions, hotels, flights, and restaurants in their vicinity. A mobile travel app can also provide tourists with a platform for booking hotels, air tickets, and tickets for visits to scenic areas.

In this study, we define compatibility as the degree to which consumers consider using a mobile travel app as a good fit for their travel-related needs and preferences. Compatibility reflects consumer perceptions of whether a mobile travel app satisfies their needs and preferences. The task technology model can be fitted to determine the relationship between task characteristics and technological characteristics that influence consumers’ evaluations of product performance and their satisfaction with that product [33].

Customer engagement is influenced by attitudinal factors [20]. A higher level of compatibility in the mobile travel app should be related to a stronger level of customer engagement. Fang et al. [24] empirically examined the influence of compatibility on psychological engagement. Kim et al. [24] report that compatibility is positively related to mobile app engagement. Therefore, we expect that compatibility positively contributes to mobile travel app engagement. Thus, we propose the following hypotheses.

H2a. Compatibility is positively related to cognitive engagement with a mobile travel app.
H2b. Compatibility is positively related to affective engagement with a mobile travel app.
H2c. Compatibility is positively related to behavioral engagement with a mobile travel app.

#### 2.2.3 UI attractiveness

UI attractiveness is defined here as the aesthetic attractiveness of a travel app based on UI design factors such as color, functional layout, and style. An attractive UI design is regarded as a powerful tool for attracting the user’s attention, improving user-technology interactions, and creating immersive and compelling experiences [34–36]. UI attractiveness is critical for creating a positive interactive experience and for promoting psychological engagement [37]. A lack of UI attractiveness leads to a reduction of one’s willingness to interact with the product [38].

An app with an attractive UI design arouses customers’ interest and makes them more willing to pay attention to the company-related information transmitted by the app. A favorable visual experience stimulates positive emotions in the user, which can motive him or her to further engage with the app and to utilize it more frequently. An attractive mobile travel app UI design affects the level of its users’ psychological engagement [24]. Therefore, we expect that an attractive mobile travel app UI design will positively influence customer engagement. We propose the following hypotheses.

H3a. UI attractiveness is positively related to cognitive engagement with a mobile travel app.
H3b. UI attractiveness is positively related to affective engagement with a mobile travel app.
H3c. UI attractiveness is positively related to behavioral engagement with a mobile travel app.

### 2.3 Purchase intention

“Purchase intention” refers to the potential for customers to purchase travel-related products provided by a mobile travel app [39]. Cognitive engagement with a mobile travel app reflects the degree to which consumers focus on the app. When consumers pay more attention to the information provided by the app, their purchase intention tendency becomes stronger. Affective engagement with a mobile travel app reflects the extent to which a consumer is affected by the app. Customers with a high level of affective engagement feel more connected to the app and are more inclined to make purchases through the app.

Behavioral engagement with a mobile travel app is the degree to which customers expend energy, effort, and time on the app. It reflects the frequency of interactions between customers and the app as well. A higher frequency of use makes the consumer more likely to purchase products through the app. Previous studies have shown that customer engagement affects behavioral intentions in the context of integrated resorts [40]. Thus, we expect that customer engagement with a mobile travel app is an influential factor leading to purchase intention. We propose the following hypotheses.

H4a. Cognitive engagement with a mobile travel app is positively related to purchase intention.
H4b. Affective engagement with a mobile travel app is positively related to purchase intention.
H4c. Behavioral engagement with a mobile travel app is positively related to purchase intention.

### 2.4 Model construction

**Fig 2:**
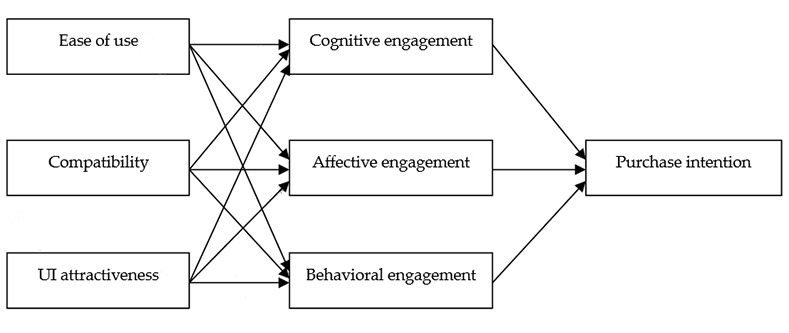
Theoretical model.

Based on the S-O-R model, we argue that travel app attributes may influence mobile travel app engagement and lead to purchase intention. Our theoretical model is shown in Fig 2.

## 3 Research design

### 3.1 Measurement development

The questionnaire used in this study includes two sections: construct items and a demographic profile. The items we used to measure the variables were adapted from previous studies and slightly modified to fit the travel app context [13,24,39,41,42]. Items for measuring ease of use [41,42], compatibility [41,42], and UI attractiveness [24], were based on previous studies. Items for measuring customer engagement were adapted from Hollebeek et al. [13]. Items for measuring purchase intention behavior were adapted from Zhang et al. [39]. All variables were measured on a 5-point Likert-type scale ranging from “strongly disagree” (1) to “strongly agree” (5).

Some of the items we used were developed in English, while all participants in this study are Chinese. We conducted a translation and back-translation procedure to ensure equivalence between the original English and Chinese versions of the items. A bilingual scholar first translated English items into Chinese, then another bilingual scholar back-translated the Chinese items into English. All authors and research assistants discussed the translations to resolve any discrepancies. We pre-tested the questionnaires with 100 samples. Three academics checked the questionnaire and confirmed the validity of the instrument. We made some minor adjustments to the wording of some items before the questionnaire was finalized and distributed.

### 3.2 Data collection and sample

We used the internet survey website “Questionnaire Star”, which is available on China’s largest online survey platform, to collect data. The formal questionnaire for this study collects sample data based on individual social relationships; samples were collected by “snowballing” from one participant throughout his or her circle of friends by referral. In the Chinese context, conducting a questionnaire through personal social relationships can better ensure respondents’ cooperation, thereby ensuring quality questionnaire responses with a maximum recovery rate.

The survey objects are strictly controlled to ensure the authenticity and reliability of the collected data. Non-experienced users were screened by asking them, “Have you ever used the mobile travel app experience?” We first explained the purpose of this study and the confidentiality of the responses. After the interviews gave their verbal consent, they were asked to complete the questionnaire. Permission was obtained from the parents of any participants under 18 years of age. The questionnaire was administered between January 7 and January 12, 2020. A total of 330 questionnaires were collected, 27 invalid questionnaires were excluded, and 303 valid questionnaires were retained.

Of the 303 respondents, 41.6% are male and 58.4% are female. More than half of the respondents (61.4%) were between 18 and 30 years old at the time of their participation. Most respondents (88.1%) held a three-year or four-year Bachelor’s degree and the second largest portion of them (11.9%) had a high school or technical secondary school diploma. The most frequent income-question response (30.7%) was 3001–5000 RMB per month, followed by 1000–3000 RMB and 5001–8000 RMB (20.8%) monthly income. The top three mobile travel apps in China are Ctrip Travel (38.0%), Qunar (18.2%), and Fliggy (17.5%). The characteristics of the sample are presented in Table 1.

**Table 1:** Sample description.

Feature	classification	Number	Percentage (%)
Gender	Female	177	58.4
	Male	126	41.6
Age	<18	5	1.7
	18–30	186	61.4.
	31–45	100	33.0
	45>	12	4.0
Eduaction	Junior middle school and below	17	5.6
	High shool∕Technical school	19	6.3
	Undergradute∕Associate Degree	168	55.4
	Postgradute Degree	99	32.7
Monthly salary	<1000¥	37	12.2
	1000–3000¥	64	21.1
	3001–5000¥	93	30.7
	5001–8000¥	63	20.8
	>8000¥	46	15.2

## 4 Data analysis and results

### 4.1 Measurement model

Partial Least Squares Path Modeling (PLS-PM) was adopted to test our hypotheses in SmartPLS 2.0 software. PLS-PM is appropriate for this study as it can be applied for the estimation of complex models with relatively small sample sizes [43]. To examine the significance of our estimates, we also ran a nonparametric bootstrapping procedure. We used SmartPLS 2.0 software for all our data analyses.

Measurement model effectiveness is typically assessed according to reliability and validity. Reliability mainly reflects the stability of the model and is typically measured by the Cronbach’s alpha and composite reliability. All the Cronbach’s alpha values of the variables are greater than 0.7. The composite reliabilities, which are more suitable for PLS-SEM than Cronbach’s alpha, range from 0.850 to 0.908 [44], i.e., exceed the recommended threshold of 0.70 [45] thus reflecting a strong level of internal consistency. The measurement model statistics are given in Table 2.

**Table 2:** Measurement model statistics.

Variables	Mean	SD	Item loading	Cronbach’s alpha	CR	AVE
Ease of use (EOU)	3.99	0.67		0.834	0.901	0.752
EOU1			0.904			
EOU2			0.830			
EOU3			0.865			
Compatibility (COM)	3.80	0.64		0.755	0.860	0.671
COM1			0.843			
COM2			0.799			
COM3			0.816			
UI attractiveness (UIA)	3.69	0.66		0.798	0.881	0.712
UIA1			0.838			
UIA2			0.826			
UIA3			0.866			
Cognitive engagement (CE)	3.68	0.66				
CE1			0.829	0.735	0.850	0.653
CE2			0.801			
CE3			0.794			
Affective engagement (AE)	3.64	0.65		0.772	0.869	0.688
AE1			0.809			
AE2			0.807			
AE3			0.871			
Behavioural engagement (BE)	3.83	0.64		0.752	0.858	0.668
BE1			0.803			
BE2			0.829			
BE3			0.819			
Purchase intention (PI)	3.63	0.73		0.848	0.908	0.766
PI1			0.866			
PI2			0.855			
PI3			0.905			

Validity mainly refers to the accuracy of a measurement model and is typically measured by convergent validity and discriminant validity. Convergent validity was examined using the average variance extracted (AVE) and indicator loadings. We calculated each construct’s AVE, ranging from 0.653 to 0.766; all are higher than the 0.5 threshold [46]. All indicator loadings are also higher than 0.5, implying convergent validity [47]. To evaluate discriminant validity, all the square roots of the AVE measures were compared with the correlations of the latent variable. All AVE measure square roots are larger than the latent variable correlations, implying discriminant validity [46]. The detailed results are shown in Table 3.

**Table 3:** The results of discriminant validity analysis.

	EOU	COM	UIA	CE	AE	BE	PI
**EOU**	0.867						
**COM**	0.637	0.819					
**UIA**	0.522	0.630	0.844				
**CE**	0.452	0.560	0.673	0.808			
**AE**	0.529	0.638	0.697	0.717	0.829		
**BE**	0.516	0.579	0.493	0.533	0.600	0.817	
**PI**	0.380	0.402	0.491	0.535	0.587	0.534	0.875

### 4.2 Structural model and hypothesis testing

The structural model shows support or lack of support for the hypothesized model. Our path analysis of the structural model is shown in Table 4. The travel app attributes appear to have different effects on the participants’ engagement with mobile travel apps. Moreover, the mobile travel app engagement appears to affect purchase intention.

**Table 4:** Path analysis of the structural model.

Path	Path name	Standardized path coefficient	P-value	Hypothesi
H1a: EOU→CE	B1	0.054	0.323	Not Supported
H1b: EOU→AE	B2	0.112	0.070	Not Supported
H1c: EOU→BE	B3	0.212	0.014*	Supported
H2a: COM→CE	B4	0.198	0.002**	Supported
H2b: COM→AE	B5	0.273	0.000***	Supported
H2c: COM→BE	B6	0.336	0.000***	Supported
H3a: UIA→CE	B7	0.521	0.023*	Supported
H3b: UIA→AE	B8	0.467	0.000***	Supported
H3c: UIA→BE	B9	0.171	0.016*	Supported
H4a: AE→PI	B10	0.181	0.014*	Supported
H4b: BE→PI	B11	0.304	0.000***	Supported
H4c: CE→PI	B12	0.255	0.000***	Supported

Notes

* P<0.050

** P<0.010

*** P<0.001.

For H1a-H1b, ease of use has no significant influence on cognitive engagement (0.054) or affective engagement (0.112). For H1c, ease of use is related to behavioral engagement (0.212*). For H2a-H2b, compatibility is related to cognitive (0.198**), affective (0.273***), and behavioral (0.336***) engagements with mobile travel apps. For H3a-H3b, UI attractiveness is related to cognitive (0.521***), affective (0.467***), and behavioral (0.171*) engagements with mobile travel apps. For H4a, cognitive engagement is related to purchase intention (0.181*). For H4b, affective engagement is also related to purchase intention (0.304***). For H4c, a significant influence was noted regarding behavioral engagement on purchase intention (0.255***). The coefficients of cognitive (48.6%), affective (55.9%), and behavioral (38.9%) engagements with mobile travel apps are explained by their predictors. Up to 41.1% of purchase intention can be explained by app attributes and customer engagement with mobile travel apps. Thus, the proposed model is well conceptualized.

### 4.3 Multi-group analysis

We grouped the data into themes according to gender, education, and income for a multi-group analysis to evaluate the differences between sub-groups. We created a distinction between low and high education (above Undergraduate/Associate), as well as between low- and high-income groups (below/above 5000 RMB per month). The results of the multi-group analysis are presented in Table 5.

**Table 5:** Results of multi-group analysis.

Path	Coefficient						
		Gender		Education		Income	
	Overall	Male	Female	Low	High	Low	High
H1a: EOU→CE	0.054	0.102	0.023	0.148	-0.005	0.172**	-0.094
H1b: EOU→AE	0.112	0.169	0.040*	0.201	0.081	0.151*	0.061
H1c: EOU→BE	0.212*	0.191	0.223*	0.110	0.133	0.304***	0.118
H2a: COM→CE	0.198**	0.153	0.210*	0.145	0.230*	0.088	0.351***
H2b: COM→AE	0.273***	0.241**	0.290**	0.263	0.260*	0.221**	0.344**
H2c: COM→BE	0.336***	0.178	0.176	0.399	0.197	0.277**	0.393**
H3a: UIA→CE	0.521***	0.499***	0.562***	0.425*	0.483***	0.545***	0.484***
H3b: UIA→AE	0.467***	0.549***	0.413***	0.450*	0.415***	0.495***	0.430***
H3c: UIA→BE	0.171*	0.178	0.176	0.310	0.419	0.153	0.195
H4a: CE→PI	0.181*	0.165	0.203*	0.256	0.156	0.145	0.270*
H4b: AE→PI	0.304***	0.276*	0.317**	0.204	0.179	0.345***	0.222
H4c: BE→PI	0.255***	0.322**	0.207*	0.434	0.313*	0.258**	0.248**

Notes

* P<0.050

** P<0.010

*** P<0.001.

In male respondents, ease of use appears to have no significant impact on engagement with mobile travel apps and purchase intention and compatibility have no significant impact on cognitive engagement with mobile travel apps. For the female respondents, ease of use is significantly associated with affective (0.040*) and behavioral (0.223*) engagements with mobile travel apps. For the less-educated, compatibility has no significant impact on engagement with mobile travel apps while for those with higher levels of education, compatibility has a positive and significant effect on cognitive (0.230*) and affective (0.260*) engagements with mobile travel apps. For the low-income group, ease of use is significantly associated with cognitive (0.172**), affective (0.151*), and behavioral (0.304***) engagements with mobile travel apps. For those in the high-income group, ease of use has no significant impact on engagement with mobile travel apps.

## 5 Discussion and conclusion

### 5.1 Summary

As mobile travel apps continue to grow more prevalent and increasingly popular, they have become an effective tool for travel companies seeking to communicate with customers and boost customer engagement. Yet, there have been relatively few studies to date on the determinants and outcomes of customer engagement with mobile travel apps. Most of the research, specifically in regards to mobile travel apps, has centered on the adoption and retention of apps [6–8]. To explore the determinants and outcomes of mobile travel app engagement, we developed a model to empirically test how app attributes (ease of use, compatibility, UI attractiveness) might influence mobile travel app engagement and in turn drive purchase intention. Finally, a multi-group analysis helps to evaluate the differences between sub-groups.

Our results show that ease of use is related to behavioral engagement with mobile travel apps. This is consistent with the findings of previous researchers who found that ease of use encourages customers to engage with m-commerce applications [27]. We did not find, however, that ease of use is related to cognitive and affective engagements with mobile travel apps. Conversely, compatibility does appear to encourage customers to engage with mobile travel apps. The relationship between compatibility and customer engagement with mobile travel apps is supported by previous studies on other types of apps [48]. Our results further suggest that UI attractiveness influences mobile travel app engagement in terms of cognitive, affective, and behavioral indicators. Likewise, previous scholars have indicated that UI attractiveness is positively associated with psychological engagement [24]. We find that customer engagement with mobile travel apps influences purchase intention; affective engagement exerted greater such effect than cognitive or behavioral engagement.

Our multi-group analysis indicated that different customers are influenced by travel attributes differently in terms of mobile travel app engagement. The results show that for the female respondents, ease of use has stronger effects on customer engagements with mobile travel apps than male respondents. For the highly educated, compatibility has a stronger effects on customer engagements with mobile travel apps than the less-educated. For the low-income respondents, ease of use has a stronger effects on customer engagements with mobile travel apps than for more affluent respondents.

In this study, we establish the determinants and outcomes of customer engagement with mobile travel apps, and make an empirical study. The results show that the antecedents of mobile travel app engagement are similar to other apps [27,48]. The results show that the three dimensions of engagement with mobile travel apps are significant antecedents of purchase intention. In particular, the results show that affective engagement has the most significant influence on purchase intention. Furthermore, a multi-group analysis shows that the attributes affecting mobile travel app engagement differ across different customer groups.

### 5.2 Theoretical implications

TAM, UTAUT, and the diffusion of innovation theory are frequently employed to explain why customers adopt mobile travel apps [6,7,8,49]. The various antecedents of mobile travel app adoption have been extensively documented, but past studies have not fully revealed why travel app attributes influence consumer engagement thus leading to purchase intention. The results of this study enhance our understanding of these effects by revealing the determinants and outcomes of customer engagement with mobile travel apps.

In this study, we expand upon previous work by Hollebeek et al. [13] on customer engagement by exploring customer engagement in the mobile travel app context, specifically. We utilized a multi-dimensional scale of customer engagement in the mobile travel app context, which deepens our understanding of multi-dimensional customer engagement theory [12–15].

In addition, although prior studies have explored antecedents of customer engagement with mobile travel apps, no previous study has fully determined antecedents multi-dimensional customer engagement with mobile travel apps or examined the effects of mobile travel app engagement on purchase intention. In this work, we established and examined a theoretical model of these factors specific to the mobile travel app context. Thus, we contribute to the customer engagement literature in general.

Our multi-group analysis shows that the effects of travel attributes on mobile travel app engagement differ in different customer groups. As Hair et al. [50] argue, a multi-group analysis can reveal the differences between sub-groups and provide new insights for further research. We grouped our respondents according to gender, education, and income to conduct multi-group analysis of the differences between the sub-groups.

### 5.3 Practical implications

We obtained some practical implications for travel-related companies and travel app developers in the process of conducting this study. Our results that improving travel app attributes induces mobile travel app engagement, which in turn influences purchase intention behavior. Travel app attributes that drive mobile travel app engagement can effectively increase customers’ purchase intention. Travel-related companies should target these attributes to encourage customers to make purchases through their apps. Mobile travel app engagement plays a critical role in inducing purchase intention, so should travel-related companies should create strong and positive connections with customers as well. These connections can be highly effective in terms of retaining customers.

Providers of mobile travel apps should prioritize their ease of use as per its critical influence on consumer engagement. App designers should minimize unnecessary functions and simplify the steps necessary to operate their apps. The tasks available on the app should be clear, actionable, and understandable. Compatibility also strongly influence mobile travel app engagement, as customers need to obtain their most needed travel products or services as simply and straightforwardly as possible. Competitive product analysis is necessary to provide customers with competitive travel products and services to meet their needs. Travel-related companies should improve their app performance attributes and optimize the functional experience to allow them to provide information (e.g., booking) as quickly and conveniently as possible.

Additionally, travel-related companies should note the importance of UI attractiveness in influencing engagement with their mobile apps. The app interface should be aesthetically pleasing and provide attractive destination information, including well-staged photographs and videos of vacation locales. In addition, travel-related companies should minimize pop-up advertisements and provide customers with a clear, “clean” interface.

### 5.4 Limitations and future work

This study contributes to the literature on customer engagement, but our work was not without limitations. First, we regarded “purchase intention” as a predictor of actual behavior, which may not be entirely accurate. Longitudinal experimental designs can be utilized in the future to ensure our results are in accordance with actual consumer behavior. Second, we mainly focus here on app features and ignore the effects of personality characteristics and social influence on mobile travel app engagement. In the future, personality factors (such as consumers’ innovativeness or personal traits) and social influences (e.g., friends’ recommendations) can be incorporated into the proposed model. Finally, our research sample comes from China, and the antecedents and consequences of mobile travel app engagement may depend on the cultural background. It is uncertain if we can extend our conclusion to other cultural backgrounds. Further research is necessary using random samples from other countries to generalize our conclusions.

## Supporting information

S1 File Measurement scales of each variable. (DOC)

S1 Data (XLSX)
