# Supplementary material for: Examining the antecedents and consequences of mobile travel app engagement
Source: PLoS One. 2021 Mar 12;16(3):e0248460. doi: 10.1371/journal.pone.0248460 (PMC7954346; doi:10.1371/journal.pone.0248460)
Supplement: S1 File — (DOC) [file pone.0248460.s001.doc]

Appendix A

| **Variables** |  | **Items** |
| --- | --- | --- |
| Ease of use | EOU1 | This travel app is easy to use. |
|  | EOU2 | Learning how to use this travel app is easy for me. |
|  | EOU3 | I would imagine that most people would learn to use this app very quickly. |
| Compatibility | COM1 | This travel app is compatible with my travel p References. |
|  | COM2 | This travel app suits my travel needs. |
|  | COM3 | This travel app ﬁts well with my travel needs. |
| UI attractiveness | UIA1 | The interface design of this travel app is beautiful. |
|  | UIA2 | The interface design of this travel app is aesthetically pleasing. |
|  | UIA3 | The interface design of this travel app is visually appealing. |
| Cognitive engagement | CE1 | This information of the travel can attract my attention |
|  | CE2 | This information of the travel can stimulate my interest |
|  | CE3 | This information of the travel can get me to think a lot |
| Affective engagement | AE1 | When I use the travel app, I feel very happy |
|  | AE2 | When I use the travel app, I feel very positive. |
|  | AE3 | When I use the travel app, I feel very good. |
| Behavioral engagement | BE1 | I spend a lot of time using the travel app, compared to other travel apps |
|  | BE2 | Whenever I’m using the travel app, I usually use this travel app. |
|  | BE3 | The travel app is one of the travel apps I usually use. |
| Purchase intention | PI1 | I intend to purchase the travel products recommended by the travel app |
|  | PI2 | I will consider purchasing travel products recommended by the travel app |
|  | PI3 | I may purchase travel products recommended by the travel app. |
